# Stability-indicating spectrophotometric quantification of safinamide in the presence of its possible degradation product

**DOI:** 10.1038/s41598-024-83195-9

**Published:** 2025-01-06

**Authors:** Ebraam B. Kamel, Omar M El-Abassy

**Affiliations:** Pharmaceutical Chemistry Department, Faculty of Pharmacy, Egyptian Russian University, Badr City, 11829 Cairo Egypt

**Keywords:** Safinamide, Stability-indicating, Parkinson’s disease, Dual wavelength, Fourier self-deconvolution, Chemistry, Analytical chemistry

## Abstract

In recent times, a truly exquisite pharmaceutical marvel has graced the world of medicine, known as Safinamide (SAF). This opulent creation has been specifically tailored to cater to the needs of individuals afflicted with Parkinson’s disease (PD), an esteemed neurological condition renowned for its regal ability to impede motor skills, coordination, and equilibrium. It is highly improbable that degradation products of pharmaceutical components would significantly compromise efficiency and safety of a drug during its shelf life. Pharmaceutical analysis requires a variety of stability tests to be conducted under distinct conditions. As a result, there was an increased need for the development of an analytical methodology capable of reliably separating and quantifying degradants and impurities that might be found in pharmaceuticals. In this study, we have developed two efficient and straightforward spectrophotometric methodologies for the concurrent estimation of SAF and its degradation product (SAF DEG), which is the main acid hydrolysis product. The confirmation of degradation product build-up by the use of several analytical techniques, including infrared spectroscopy (IR), and mass spectrometry (MS) investigations. The present methodologies have been validated for linearity within the concentration range of 5–30 µg/ml for SAF, and 5–15 µg/mL, 2–15 µg/ml for SAF DEG for fourier self-deconvolution (FSD) and dual wavelength (DW) methods, respectively. The originality of these techniques lies in their status as the first stability-indicating spectrophotometric procedures that are both environmentally friendly. Moreover, the process of obtaining pure SAF DEG offers substantial economic benefits by obviating the need to acquire a costly constituent. The use of intelligent techniques was employed to analyze the pharmaceutical dosage form, potentially offering significant advantages to the pharmaceutical industry.

## Introduction

The presence of drug degradation products, even in small amounts, can impact the effectiveness and safety of the drug throughout its shelf life. Pharmaceutical analysis necessitates comprehensive stability testing across various conditions. By conducting these studies, we can gain a deeper understanding of the inherent stability of the active substance and make informed predictions about the potential degradation pathways that are most likely to occur. As a result, there is a significant need to establish a reliable analytical technique to isolate and accurately measure drug degradation products^1,2^.

One of the leading causes of mortality and sickness globally is neurodegenerative disease, which mostly impacts older individuals^3^. Parkinson’s disease (PD) is a significant neurological condition that is positioned second concerning prevalence among neurological diseases, just behind Alzheimer’s disease (AD). The breakdown of dopaminergic neurons in the brain can lead to a variety of motor and non-motor abnormalities, which are associated with Parkinson’s disease.

**Fig. 1:**
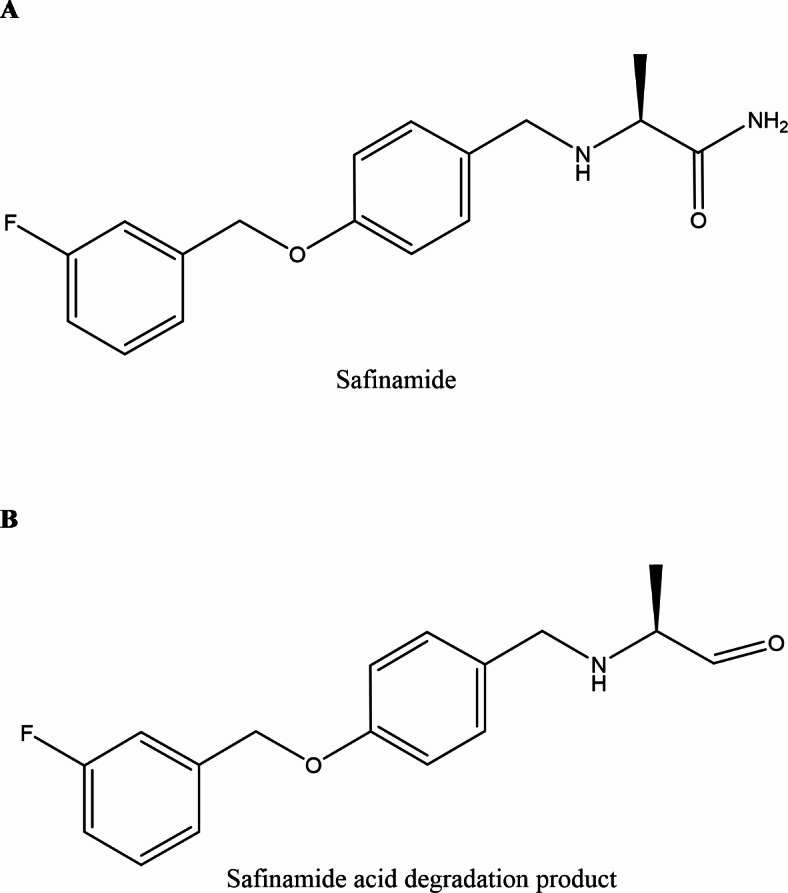
(**a**) Chemical structure of SAF and (**b**) Chemical structure of SAF DEG.

Safinamide (SAF), showed in Fig. 1a, is an important improvement to the treatment of PD and an alpha-aminoamide (MAO-B) inhibitors. Chemically, it is known as (S)-2-((4-((3-Fluorobenzyl ) oxy ) benzyl ) amino) propanamide methanesulfonate and it has a molecular weight of 302.35 gm/mole^4^ As a “add-on therapy to a stabilized dosing of levodopa alone or in combination with additional PD medications in middle to advanced stage fluctuating patients,” it was granted approval by the European Union and the FDA in February 2015 and March 2017, respectively^3,5^. Peer-reviewed publications have published a large number of papers on the assessment of SAF either by itself or in conjunction with other drugs, such as the following: thermal examination^6^, HPTLC^7,8^, HPLC^7,9–15^, UPLC^7^, electrochemistry^16,17^ and spectroscopy^18,19^.

Pharmaceutical analysis needs considerable stability tests under a wide range of conditions. These investigations provide the opportunity to predict the most likely degradation pathways and to ensure the active substance’s inherent stability features. Because of this, there was an increase in the need for developing an analytical technique that could reliably separate and quantify degradation products and/or impurities of pharmaceuticals^20–24^. Therefore, in the present investigation, SAF and SAF DEG which known chemically as (S)-2-((4-((3-fluorobenzyl)oxy)benzyl)amino)propanal and has molecular weight of 287.33 gm/mole were determined simultaneously.

Consequently, we worked to create environmentally acceptable spectrophotometric techniques for the simultaneous measurement of SAF and the outcome of its degradation in raw materials, mixtures synthesized in the laboratory, and pharmaceutical dosage form (safinozol^®^ tablets).

Because of its creativity, ease of use, cost- and time-saving benefits, and simplicity, our work has a distinct uniqueness and excellence.

## Experimental

### Instrument

The spectra manager software from Jasco was used to operate the UV spectrophotometer on a desktop PC that was ACER compatible. Quartz cells with a 1.0 cm diameter was used to handle absorption spectra of reference and test solutions in the wavelength range of 200–400 nm.

### Materials, reagents and solvents

With a certified purity of 99.80%, October Pharma, Cairo, Egypt, has kindly donated SAF. Safinozol^®^ tablets were produced and donated by October Pharma, Cairo, Egypt (Batch No. 15FF407Z, labeled as having 100 mg SAF). We used ethanol (HPLC grade, Sigma, Germany). Sodium hydroxide and hydrochloric acid (Sigma, Germany) were used for producing SAF DEG.

### SAF acid degradation product Preparation (SAF DEG)

An exact weight of 100 mg of SAF was blended with 25 mL of 1 N HCl dissolved in ethanol, and the mixture underwent 6 h reflux at a temperature of 90 °C. To guarantee total degradation, 0.5 mL of the refluxed solution was diluted further with diluent and applied to a plate of TLC using a mobile phase composed of chloroform: methanol: ammonia in a ratio of (2:4:0.5, v/v/v). Upon examination of the TLC plate, one spots were observed for SAF DEG (Rf = 0.85). After cooling and neutralizing the solution with 2 N NaOH, it was dried by evaporation. Hot ethanol was used to purify the residue, and then filtered and dried till evaporation. Employing IR and MS spectroscopy, the acid degradant has been located, and its structure was determined.

### Stock solutions

#### Standard solutions

To produce the standard stock solution, SAF was dissolved in ethanol to a concentration of 1 mg/ml. The stock solution was properly diluted with ethanol to provide a standard working solution of SAF at a concentration of 100 µg/mL. The standard solutions were made fresh and were stable in the refrigerator for at least four weeks.

#### Acid degradation product solutions

A solution of the acid degradation product (SAF DEG) at a concentration of 1 mg/mL was produced by accurately weighing 100 mg of SAF DEG and dissolving it in ethanol within a volumetric flask with 100 mL volume. Both SAF and SAF DEG standard working solutions concentration was 100 µg/mL.

#### Development of synthetic mixtures in the laboratory

Accurate aliquots of both SAF and SAF DEG were carefully measured into two distinct 10 mL volumetric flasks. Each flask was then filled to the mark with ethanol and thoroughly mixed.

### Procedures

#### Establishing linearity intervals and calibration graphs

To obtain concentrations ranging from 5 to 30 µg/mL for SAF. For SAF DEG, from 5 to 15 µg/mL and from 2 to 15 µg/mL for FSD and DW methods, respectively. Aliquots were transferred into two sets of 10 mL volumetric flasks separately from the standard working solutions of SAF (100 µg/mL) and SAF DEG (100 µg/mL). Ethanol was then used to bring the total volume to the mark. The absorption spectra of the resulting solutions for both compounds were recorded between 200 and 400 nm, with ethanol serving as the blank.

#### Dual wavelength spectrophotometric method (DW)

The calibration curve was constructed by measuring the absorbance difference between 223.6 nm and 228 nm, which was then plotted against various concentrations of SAF. For the quantification of SAF DEG, the absorbance values at 220 nm and 233 nm were recorded from the SAF DEG spectra, and the corresponding differences were plotted against the concentrations of SAF DEG. Regression equations were subsequently derived from both plots.

#### Fourier self-deconvolution spectrophotometric method (FSD)

The Jasco spectrum software’s Fundamental Spectrum Deconvolution (FSD) function was utilized to deconvolute the recorded spectra. Plotting the deconvoluted spectra signals against their corresponding concentrations was done for SAF at 235 nm and SAF DEG at 242.3 nm. Next, the related regression equations were utilized to calculate the concentrations of SAF and SAF DEG in both the dosage forms of SAF and their laboratory-synthesized mixtures.

#### Analysis of combinations synthesized in the laboratory

The spectra of the laboratory-synthetic combinations that were scanned earlier were processed as described in the section titled “Establishing linearity intervals and calibration graphs” for the two methods. Each analytes’s concentrations were determined using the regression equations that had been developed previously.

#### Quantification of safinozol® tablets

Ten safinozol^®^ tablets were carefully weighed, then grinded and thoroughly mixed. A portion of the resulting powder, equivalent to 100 mg SAF, was weighed and moved to 100 mL flask. Thirty milliliters of ethanol were added as a diluent, and the mixture was sonicated for 30 min. Filtration was performed using a dry funnel and filter paper, the initial few milliliters being removed. The volume was then adjusted to the 100 mL mark with ethanol and mixed thoroughly. This process yielded sample stock solutions with a concentration of 1 mg/mL of SAF. Subsequently, to prepare sample working solution of SAF at 100 µg/mL, appropriate dilution of the 1 mg/mL stock solution was made. Various aliquots were then transferred to volumetric flasks with 10 mL volume and diluted with the diluent within the previously established linearity range. The procedures detailed in the section “Establishing linearity intervals and calibration graphs” were followed. The standard addition technique was employed to verify the correctness of the procedure, and the related regression equations were utilized to ascertain the pure drug’s concentration.

## Results and discussion

### Acid degradation product structure elucidation (SAF DEG)

**Fig. 2:**
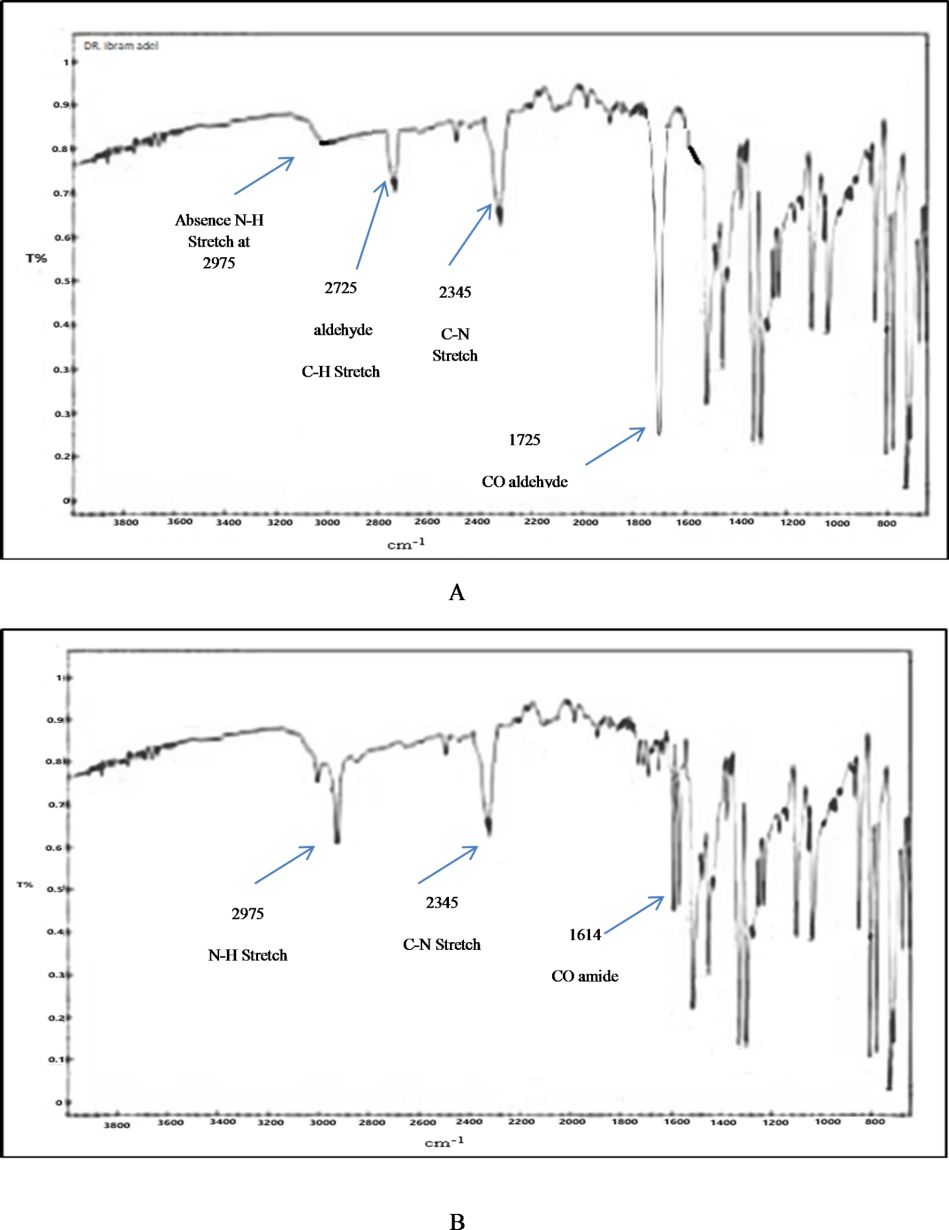
(**a**) IR spectrum of SAF DEG (**b**) IR spectrum of SAF intact drug showing the appearance and disappearance of some groups confirming the structure of SAF DEG.

**Fig. 3:**
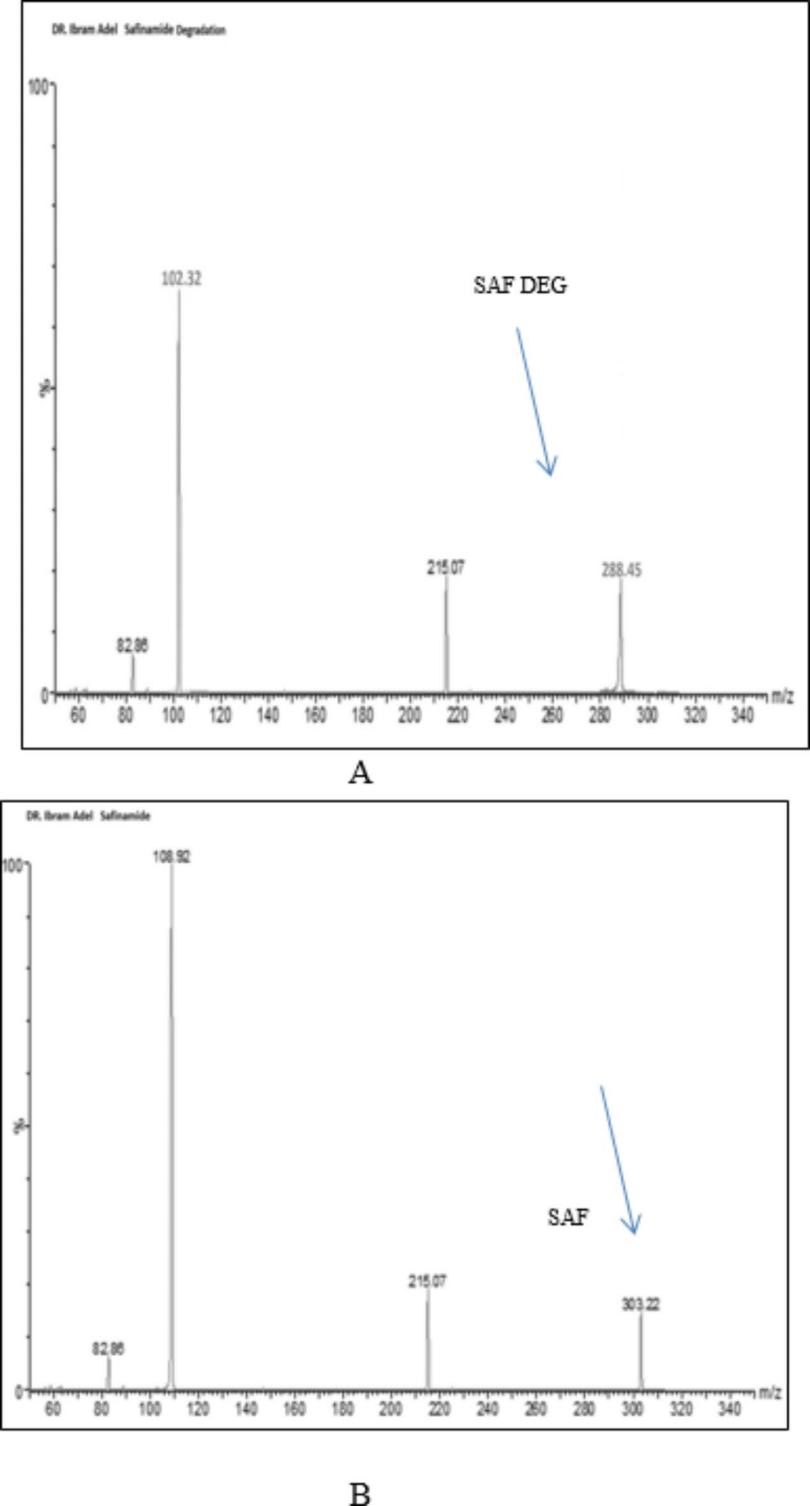
(**a**) MS spectrum of SAF DEG m/z 288.45 (**b**) MS spectrum of SAF intact drug m/z 303.22 confirming the complete degradation of the intact drug SAF to SAF DEG.

SAF was subjected to reflux with 1 N ethanolic HCl for six hour at 90 °C to ensure total degradation and maximize the production of the resulting SAF DEG (Fig. 1b). After refluxing, neutralization of the solution was carried out and then evaporated. SAF DEG was then developed through purification and extraction in hot ethanol, and then evaporated to become dry. TLC was employed to characterize the developed yield, revealing a new band corresponding to SAF DEG (R_f_ = 0.85) was identified, which is distinct from the intact drug’s profile (R_f_ = 0.65). SAF DEG IR spectrum (the acid degradation product), (Fig. 2a), illustrated the disappearance of N-H stretching band at 2975 cm^− 1^ which found in SAF’s IR spectrum (Fig. 2b) and also disappearance C-O amide band at 1614 cm^− 1^ that appeared as C-O aldehyde at 1725 cm^− 1^. Also, appearance of N-H stretches of aldehyde at 2725 cm^− 1^ in SAF DEG is evidence that SAF turned over to SAF DEG. Moreover, mass spectroscopy, M^+^ at 288.45 which is identical to the molecular weight of SAF DEG (Fig. 3a). Also, mass spectroscopy, M^+^ at 303.22 which matches SAF’S molecular weight (Fig. 3b). It is confirmed by the results of the IR and MS spectra analysis that a pure version of SAF DEG was achieved. Comparing this accomplishment to the purchase of an expensive ingredient, significant cost savings are realized. Furthermore, the synthesized SAF DEG can be utilized in future studies aimed at treating Parkinson’s disease.

### Methods optimization

#### Dual wavelength spectrophotometric method (DW)

**Fig. 4:**
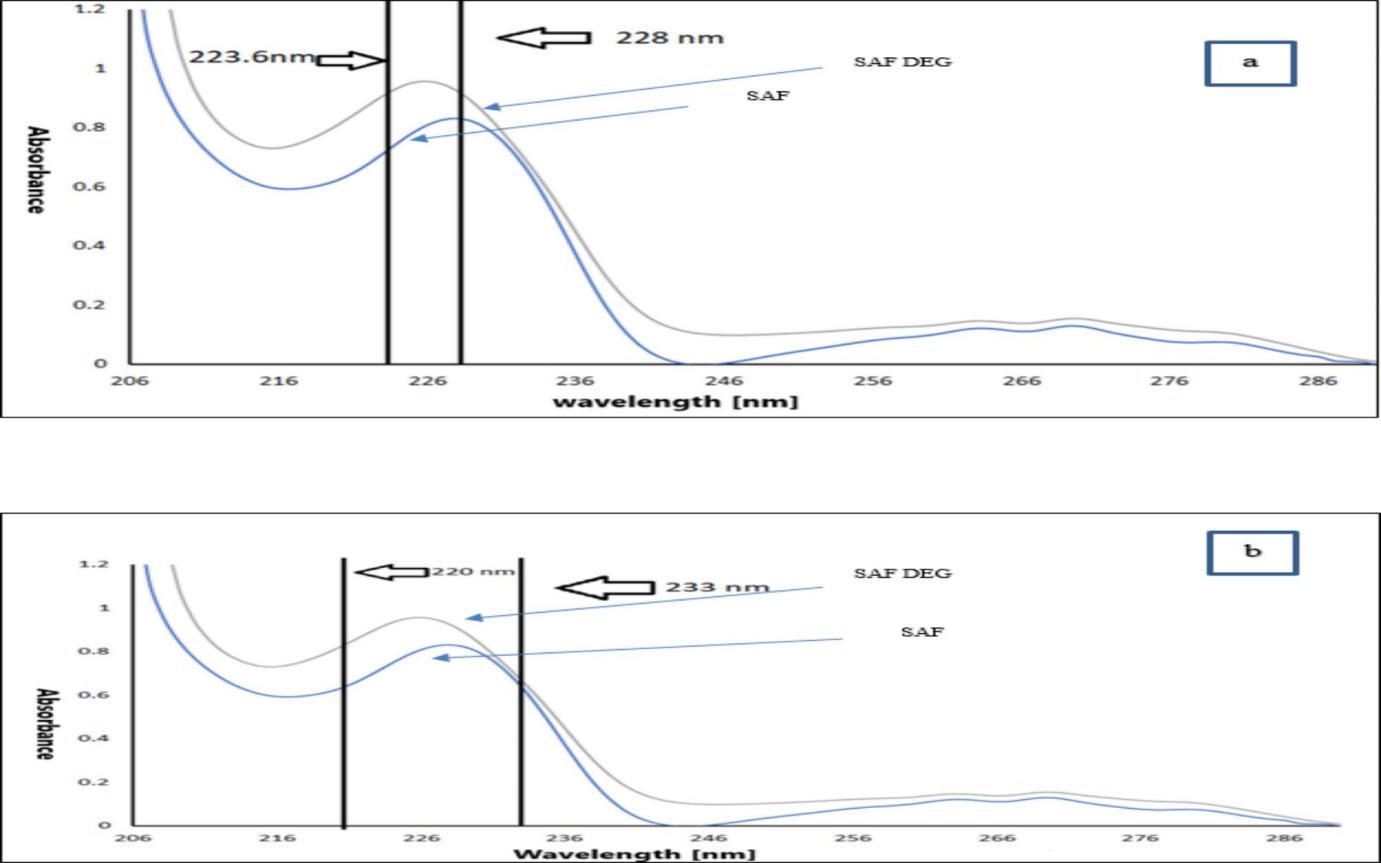
(**a**) Absorption spectrum of SAF measuring the absorbance difference between 223.6 and 228 nm at which the absorbance difference of SAF DEG is zero and (**b**) Absorption spectrum of SAF DEG measuring the absorbance difference between 220 and 233 nm at which the absorbance difference of SAF is zero.

The core concept of this method is to identify a significant difference in absorption between two specific points, while ensuring that the variation in absorbance for any additional overlapping component within the mixture is negligible or virtually non-existent^25^. To estimate SAF in a mixture with SAF DEG, multiple pairs of wavelengths were analyzed at which the absorbance difference between SAF and SAF DEG was found to be zero. By calculating the absorbance difference between 223.6 and 228 nm, a satisfactory linear response for SAF was achieved (refer to Fig. 4a). The absorbance difference between these wavelengths increases with higher concentrations of SAF, without any interference from SAF DEG. Conversely, SAF DEG was quantified without interference from SAF by measuring the difference in absorbance values between 220 and 233 nm. This difference rises as the concentration of SAF DEG rises (see Fig. 4b).

#### Fourier self-deconvolution spectrophotometric method (FSD)

**Fig. 5:**
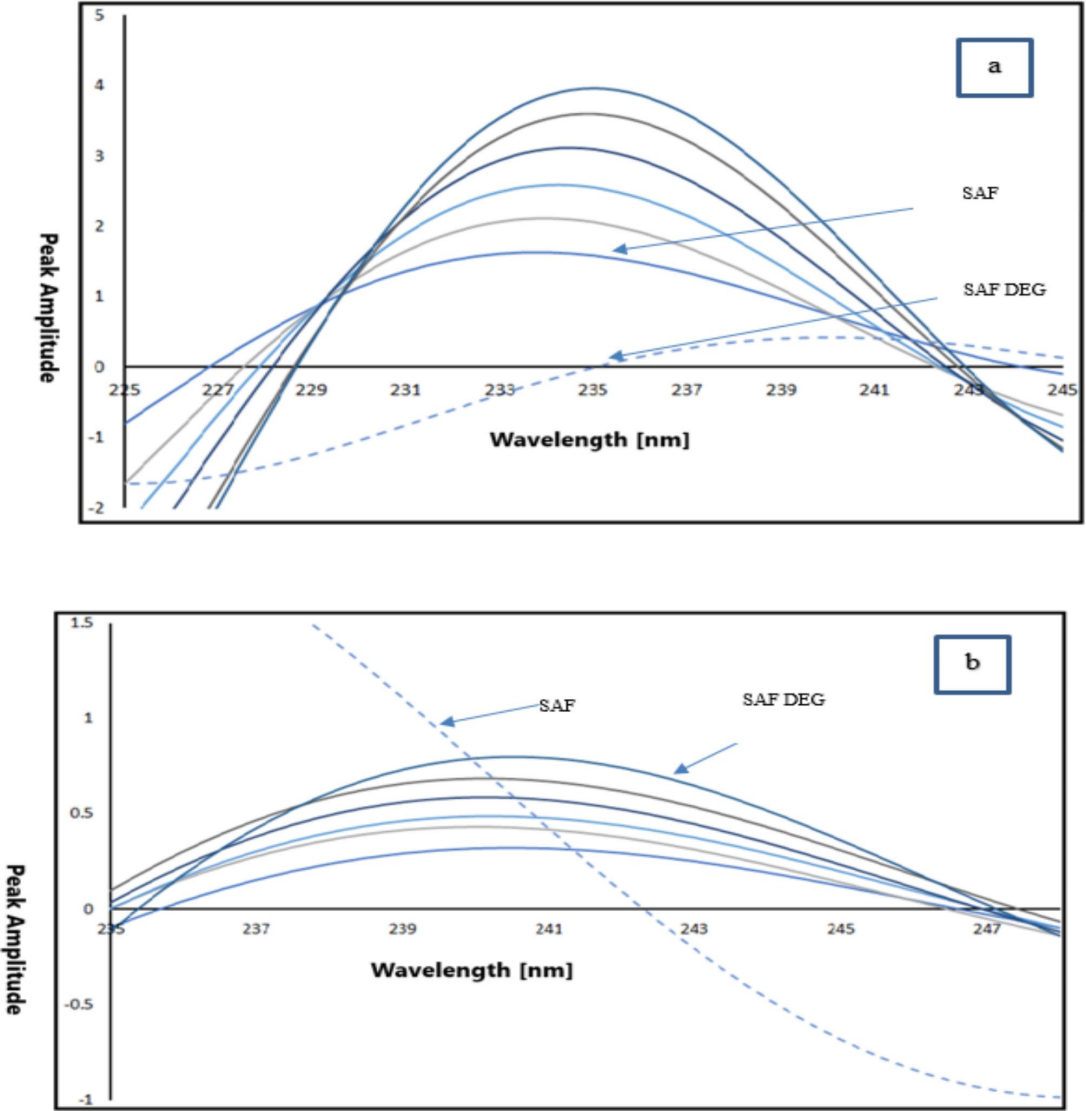
(**a**) Deconvoluted spectrum of SAF measured at 235 nm at which SAF DEG is zero-crossing and (**b**) Deconvoluted spectrum of SAF DEG measured at 242.3 nm at which SAF is zero-crossing.

Fourier self-deconvolution (FSD) is a commonly used mathematical technique for narrowing bands, which enables the resolution of overlapping spectral bands, such as those found in the spectra of a mixture of SAF and SAF DEG. The FSD methodology is employed to accurately identify the dominant sites of each band within the developed spectra of this SAF and SAF DEG mixture, which consists of many spectra that share identical bandwidths. The degree to which the deconvolved bands are narrowed (known as effective narrowing) and the impact on the signal-to-noise ratio caused by FSD are significantly influenced by the selection of filter tool used^26^. The chosen filter function significantly affects the shape of the deconvolved bands. The absorption spectra of SAF and SAF DEG can be determined using the FSD tool in Jasco spectral software. The resulting deconvoluted spectra were employed to identify SAF at 235 nm (Fig. 5a) and SAF DEG at 242.3 nm (Fig. 5b) in the presence of both substances (zero-crossing point), using an expected Lorentzian waveform with a full width at half maximum (FWHM) value of 60. Various FWHM values were tested until well-resolved spectra with consistent and reliable recoveries were achieved. The FWHM value is a critical factor to consider, as the program is not effective for resolving overlapping measured spectra that contain peaks of differing widths. Consequently, both the half-widths of the spectral bands will be considered both before and after the deconvolution process, represented by their respective FWHM values^27,28^.

### Methods validation

The ICH Specifications have been completely complied within the development of the proposed procedures. The following data express step by step complete analytical method validation as stated by ICH Q2 guidelines^29^.

### Linearity

The strong correlation coefficient of all the indicated analytes confirmed the good linearity of the current methods. Table 1 summarizes the linearity ranges for various concentrations of SAF and SAF DEG.

**Table 1:** Regression and validation parameters for spectrophotometric determination of SAF with SAF DEG.

Parameters		FSD		DWL	
		SAF	SAF deg	SAF	SAF deg
Range		5–30	5–15	5–30	2–15
Correlation coefficient		0.9993	0.9996	0.9994	0.9995
Slope		0.092	0.044	0.008	0.011
Intercept		1.187	0.060	− 0.020	− 0.017
LOD^a^		1.138	0.437	1.039	0.440
LOQ^b^		3.450	1.324	3.149	1.335
Wavelength		235 nm	242.3	223.6–228 nm	220–233 nm
Accuracy (Recovery%^c^ ± SD)		100.528 ± 0.655	100.140 ± 0.352	99.544 ± 0.112	99.981 ± 0.275
Precision	Intraday^d^	0.218	0.726	0.516	0.602
%RSD	Interday^d^	0.689	0.887	1.034	0.713

^a^3.3*SD of intercept/slope.

^b^10*SD of intercept/slope.

^c^Average of nine estimations.

^d^RSD% of nine estimations.

### Detection and quantitation limits

Calibration curve slope and intercept standard deviation were used to determine limit of detection (LOD) and limit of quantitation (LOQ). Table 1 displays the values of LOQ and LOD for both SAF and SAF DEG. The LOD and the LOQ were calculated according to ICH guidelines from the following equations:

$$\begin{gathered} {\text{LOD}}\,=\,{\text{3}}.{\text{3}}\sigma /{\text{S}} \hfill \\ {\text{LOQ}}\,=\,{\text{1}}0\sigma /{\text{ S}} \hfill \\ \end{gathered}$$

where σ is the standard deviation of y-intercepts of regression lines and S is the slope of the calibration curve.

### Accuracy

In triplicate, three concentration levels of SAF (10, 15, and 25 µg/mL) and SAF DEG (6, 10, and 12 µg/mL) were analyzed. Their found concentrations and average percentage recoveries (R%) were then computed. Table 1 provides a summary of the findings and demonstrates the precision of the current techniques.

### Precision

#### Intraday

Three different concentrations of SAF (10, 15, and 25 µg/mL) and SAF DEG (6, 10, and 12 µg/mL) were analyzed 3 times in the same day by the suggested methods. RSD % values were calculated for each method as shown in Table 1.

#### Interday

The previously chosen three different concentrations were analyzed by the suggested methods over three different days and %RSD values were calculated for each method (Table 1).

### Specificity

By assessing laboratory-synthesized mixtures comprising various percentages of SAF and SAF DEG, the selectivity of the suggested approaches was assessed, as shown in Table 2. Selectivity was validated using the standard addition procedure by adding known quantities of SAF and SAF DEG to pre-analyzed Safinozol^®^ solution at three distinct concentrations. There was no interference from excipients. As shown in Table 3, the mean recovery percentage (% R) and % standard deviation percentage (% SD) were computed.

**Table 2:** Determination of laboratory prepared mixtures of SAF and SAF DEG by the proposed spectrophotometric methods.

Methods	FSD		DWL	
Concentration (µg/mL)	Found %^a^			
	SAF	SAF deg	SAF	SAF deg
SAF: SAF deg				
7:15	99.594	101.270	100.752	100.093
15:7	99.784	99.731	99.965	99.142
10:10	101.167	101.455	100.788	99.666
25:5	100.004	100.024	98.978	100.507
30:8	99.700	100.605	99.443	99.184
Mean ± SD	100.050 ± 0.642	100.617 ± 0.752	99.985 ± 0.797	99.719 ± 0.587

^a^Average of three determinations.

**Table 3:** Determination of safinozol ^®^ tablets by the proposed methods and application of standard addition technique.

Drugs	SAF	
Methods	FSD	DWL
Pharmaceutical dosage form^a^ (found% ± SD)^b^	100.862 ± 0.971	101.114 ± 0.898
Standard Addition (recovery% ±SD)^b^	100.076 ± 1.026	100.463 ± 0.573

^a^Safinozol tablets batch No. 15FF407Z, labeled as having 100 mg SAF.

^b^Average of three determinations.

### Statistical analysis

Table 4 provides a statistical comparison of the results obtained from the proposed methods against the reference method^19^. The observed t and F values were lower than the theoretical values, suggesting that the established and reported procedures demonstrate similar accuracy and precision (as indicated in Table 4). These results confirm that the established methods can quantitatively measure SAF with high precision and considerable accuracy.

**Table 4:** Statistical comparison of safinozol ^®^ tablets results between the proposed spectrophotometric methods and published method.

Parameters	SAF		
	Proposed methods		Reported method^19^
	FSD	DWL	
Mean	100.093	99.839	101.324
SD	1.288	0.901	1.254
n	5	5	5
Student’s t-test (2.306)^a^	1.53	2.15	
F-value (6.39)^a^	1.05	1.94	

^a^The theoretical values of t and F at *P* = 0.05 are (2.306) and (6.39), respectively.

### Assessment of greenness of the analytical methods

Environmentally friendly chemistry is extensively adopted in chemical laboratories, needing specific assessment techniques for precisely assessing the environmental effect of chemical operations. Several approaches may be used to assess greenness. To validate the environmental friendliness of analytical procedures, four key factors are considered: chemical amounts and risks, notable energy consumption, workplace hazards, and waste generation. The environmental sustainability of the suggested approach was evaluated using the National Environmental Methods Index (NEMI), the Analytical Greenness Calculator (AGREE), and the Green Analytical Procedure Index (GAPI).

**Fig. 6:**
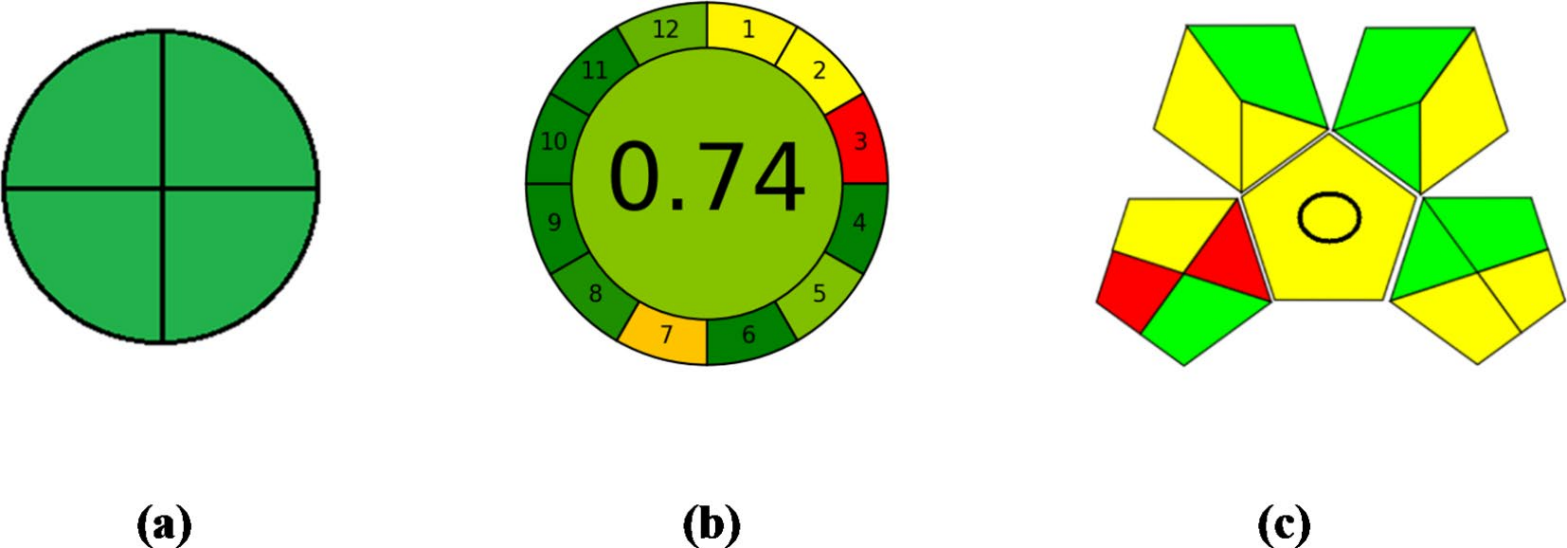
Green assessment of the proposed spectrophotometric methods using different tools, (**a**) NEMI, (**b**) AGREE and (**c**) GAPI.

The first approach primarily relies on the National Environmental Methods Index (NEMI)^30^, which illustrates environmental impact through a pictogram divided into four quadrants. NEMI is a qualitative tool that uses pictograms to indicate the use of toxic or corrosive chemicals, as well as the potential for substantial waste generation. In the proposed spectroscopic approaches (Fig. 6a), the four quadrants will be marked in green, indicating that the solvent used (ethanol) is neither persistent, bioaccumulative, nor toxic (PBT), and is not classified as hazardous. Furthermore, the pH levels of the processes range from 2 to 12, and the waste produced is less than 50 g.

The AGREE^®^ software serves as the foundation for the second system. It features a clock-shaped graph founded on the 12 principles of Green Analytical Chemistry (GAC)^31–33^. The compatibility of the analytical methodology with the principles of green analytical chemistry is assessed on a color scale (red–yellow–green), with each section representing a different principle. The center of the AGREE graph displays both a color denoting the overall rating and a numerical score between 0 and 1, indicating the strength of the rating for every segment. The software evaluates the environmental effect of various factors, including reagent hazard, waste production, energy usage, labor demand, and the level of automation. The goal is to minimize environmental harm and promote more sustainable production methods. A final score of 0.74, which reflects the environmental sustainability of the approach, was computed via determination of specific values to the parameters used in the study, as shown in (Fig. 6b).

The Green Analytical Procedure Index (GAPI)^34–36^ was also employed to assess the greenness of the analytical methods. GAPI comprises fifteen distinct areas represented by five pentacle shapes, which evaluate the ecological effect of each stage in the analytical technique, from sample development to apparatus and waste generation and treatment. These parameters are visually represented on the pentagram, colored green, yellow, or red to signify minimal, moderate, or high environmental impact, respectively.

In the established spectroscopic methods, the inline preparation of the sample eliminated the need for storage. The samples were prepared directly without any extraction procedures, utilizing green solvents and without supplementary treatments. Sections 1 and 3 were marked red due to offline sampling and transportation required for the sample. Sections 4,6,7 and 11 were marked yellow due to the standard storage conditions employed, the microextraction method used for the sample, the use of a green solvent and the high flammability score of ethanol (3) according to NFPA respectively (as illustrated in Fig. 6c).

Furthermore, Sect. 5 was highlighted in yellow to indicate the quantification methods employed in the sample preparation (filtration) for the pharmaceutical dosage form. Sections 14 and 15 were also marked yellow because the waste generated about 10 mL and degradation of ethanol (Fig. 6c).

Overall, as depicted in Fig. 6c, the GAPI results demonstrate a favorable greenness for the methods evaluated.

In a nut shell, it was observed that the evaluation instruments employed for the systems’ metrics were complementary to each other in profiling the sustainability of the proposed methodologies. Thus, they can be applied for the routine analysis of the selected drugs, yielding a positive environmental impact, reduced use of hazardous reagents, and minimized risk of toxic effects^34^.

## Conclusion

The proposed methodologies offer simple, sensitive, and accurate spectrophotometric methods for the simultaneous determination of SAF and SAF DEG, which might be a result of acid degradation, using inexpensive and ecologically acceptable solvents. SAF is an important improvement to the treatment of PD and an alpha-aminoamide (MAO-B) inhibitor. The overlapping spectra of SAF and SAF DEG as well as other complicated combinations may be analyzed with a variety of tools using the spectrophotometric approaches that have been presented. The AGREE calculator, GAPI index, and NEMI approach were used to evaluate the environmental effect of these techniques. The presented methods were validated according to ICH recommendations, showing excellent results. Also, the results of developed methods compared statistically to previously published method and there was no statistical difference.

## Data Availability

The datasets used and/or analyzed during the current study are available from the corresponding author on reasonable request.
